# Pathologically noninvasive cancer predictors and surgical procedure for peripheral lung cancer

**DOI:** 10.1111/1759-7714.14749

**Published:** 2022-11-22

**Authors:** Hiroyuki Tsuchida, Masayuki Tanahashi, Eriko Suzuki, Naoko Yoshii, Takuya Watanabe, Shogo Yobita, Suiha Uchiyama, Kensuke Iguchi, Minori Nakamura, Takumi Endo

**Affiliations:** ^1^ Division of Thoracic Surgery, Respiratory Disease Center Seirei Mikatahara General Hospital Shizuoka Japan

**Keywords:** C/T volume ratio, peripheral small size non‐small cell lung cancer, preoperative pathological predictor of noninvasive cancer, Synapse Vincent

## Abstract

**Background:**

In this retrospective study, based on recent studies reporting the superiority of sublobar resection to lobectomy for peripheral small size non‐small cell lung cancer (NSCLC), we investigated the optimal pathological factors for predicting noninvasive cancer and the selection of operative procedure.

**Methods:**

Patients with peripheral NSCLC of ≤2 cm who underwent surgery at our hospital between January 2010 and June 2020 were included in this study. We evaluated the relationship between pathologically noninvasive cancer and predictive factors according to the area under the curve (AUC) and accuracy, and the cutoff value was set to investigate indications for sublobar resection.

**Results:**

The comparison of the AUCs revealed that the maximum standardized uptake value and consolidation to tumor (C/T) volume ratio were better predictors than the C/T ratio. Among the three factors, the C/T volume ratio showed the best accuracy. The patients were divided into two groups (low and high) using the cutoff value of the C/T volume ratio and compared according to the surgical procedure (lobectomy vs. segmentectomy). In the low‐group, there was no significant difference in the prognosis. In the high‐group, the 5‐year recurrence‐free survival rate of the patients who received lobectomy was 87.8%, while that of patients who received segmentectomy was 75.8% (*p* = 0.08).

**Conclusions:**

The C/T volume ratio was the best preoperative pathologically noninvasive predictive factor. Sublobar resection should be performed with caution in cases with significant solid components on three‐dimensional images.

## Graphical abstract

The consolidation to tumor ratio of volume was the best preoperative pathologically noninvasive predictive factor. Sublobar resection should be performed with caution in cases with significant solid components on three‐dimensional images.

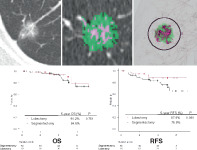

## INTRODUCTION

The current standard treatment for lung cancer is lobectomy. However, in the case of peripheral small size non‐small cell lung cancer (NSCLC), the frequency of lymph node metastasis is low; thus, it is considered a good indication for sublobar resection.^1^ In recent years, multiple large‐scale studies have been conducted using the maximum tumor diameter and consolidation to tumor (C/T) ratio as indices to determine the indications for sublobar resection.^2^, ^3^, ^4^ The results of the Japan Clinical Oncology Group (JCOG) 0802/West Japan Oncology Group (WJOG) 4607L showed that segmentectomy was superior to lobectomy in terms of overall survival (OS) for peripheral NSCLC with a maximum tumor diameter of <2 cm and solid component predominance (C/T ratio, 0.5 to 1.0),^2^ and that it had a great impact on the treatment of early‐stage lung cancer, which has been increasing in recent years.^5^ On the other hand, the rate of local recurrence (margin recurrence) in patients who receive segmentectomy is approximately twice that of patients who receive lobectomy, and mortality from other cancers was predominant in the segmentectomy group. Questions remain as to the true superiority of segmentectomy. In particular, the high risk of local recurrence with segmentectomy is a matter of great concern as a surgical treatment for early‐stage lung cancer aiming for a radical cure. Factors that have been reported to be related to local recurrence include accurate preoperative evaluation of malignancy, surgical decision‐making based on evaluation, and surgical skill. In this study, we investigated the evaluation of a preoperative malignancy and the determination of the surgical procedure.

Sublobar resection with curative intent for primary lung cancer should be indicated for a pathologically noninvasive cancer. In order to search for a more accurate index than the C/T ratio, we investigated the preoperative pathological predictors of noninvasive cancer in peripheral small size NSCLCs. In addition, a prognostic analysis was performed using the best predictive factors, and the selection of surgical procedure was examined.

## METHODS

### Patients

**FIGURE 1:**
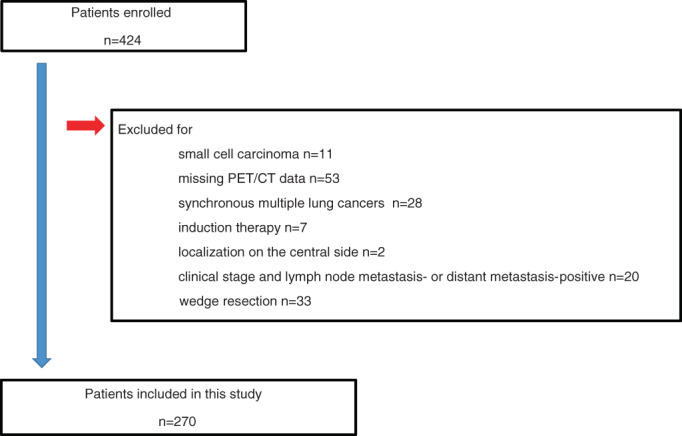
Flow diagram of the patient selection

Among 1120 primary lung cancer patients who underwent surgical treatment at our department between January 2010 and June 2020, 424 with a maximum tumor diameter of ≤2 cm were included in this study. The preoperative pathological predictors of noninvasive cancer were examined in 270 cases, after excluding patients with the following factors: small cell carcinoma, positron emission tomography (PET)/computed tomography (CT) not performed, synchronous multiple lung cancers, performance of preoperative chemoradiotherapy, central type, clinical stage with lymph node metastasis or distant metastasis, or a wedge resection had been performed. The prognosis was analyzed and the selection of the surgical procedure was examined (Figure 1).

### Definition of pathologically invasive and noninvasive carcinomas

In this study, vascular invasion, lymphatic invasion, and lymph node metastasis, which were agreed upon in JCOG0201,^6^ were used as factors for judging pathologically invasive cancers. In addition, cancers with spread through air spaces (STAS) and micropapillary positivity, pathological factors that been reported to be associated with a poor prognosis,^7^, ^8^ were considered invasive. Cancers that were negative for all five factors were defined as pathologically noninvasive. Cancers that were positive for any one factor were defined as pathologically invasive.

### Preoperative pathological factors predicting noninvasive cancer

**FIGURE 2:**
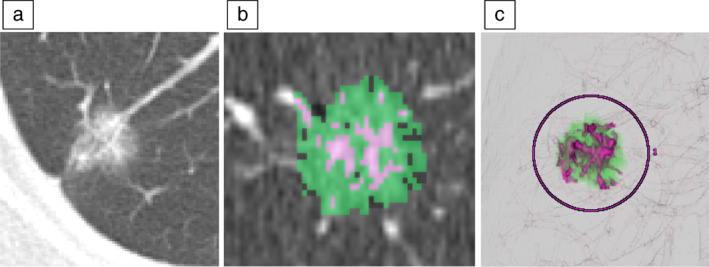
High‐resolution computed tomography (CT) (a). Ground‐glass opacities (GGO) (green) and solid (purple) regions analyzed with the Synapse Vincent software program (b) and the 3D image used for the analysis (c)

Preoperative pathological factors predicting noninvasive cancer included the conventional C/T ratio, maximum standardized uptake value (SUVmax), carcinoembryonic antigen (CEA), and reciprocal of tumor doubling time (doubling rate), as well as the volume of the entire nodule (tumor volume), volume of the consolidation part (consolidation volume), and C/T ratio of volume (C/T volume ratio) measured using the three‐dimensional (3D) Synapse Vincent analysis software program (Fuji Photo Film Co., Ltd.) (Figure 2). The doubling rate was calculated using stored data of CT performed immediately before surgery and the oldest scan in which the tumor could be confirmed. All patients underwent preoperative CT with a slice thickness of <3 mm. All CT findings were reviewed by the author.

### Volume measurement with Synapse Vincent

Volume measurement method using Synapse Vincent was performed as follows. After starting Synapse Vincent, the patient ID was entered into the system and the CT data and series were selected for the analysis. Then “Lung Analysis” or “Lung Resection Analysis” was selected on the pop‐up screen that appeared. A line was drawn so as to connect both ends of the entire nodule in an arbitrary slice of the CT image displayed after a few seconds. The software semi‐automatically recognized the nodule using these operations, and by clicking the GGN Analysis tab on the bottom right of the screen, the volume and ratio of the entire nodule and solid part below it could be checked. In this way, the measurements could be performed in just a few minutes.

### Identification of the best predictive factors and the prognostic comparison

The above‐mentioned predictors were compared using the area under the curve (AUC) determined from a receiver operating characteristic (ROC) curve with the C/T ratio as a control, and superior factors were extracted. Furthermore, in order to minimize the risk of preoperatively diagnosing an invasive cancer as a pathologically noninvasive cancer and performing sublobar resection, we referred to the analysis results of JCOG0201 and selected a maximum specificity of 0.97.^6^ The best predictor was identified by comparing the sensitivity using a set the cutoff value. Furthermore, the cutoff value of the best predictive factor was used to divide the population into two groups, and OS, recurrence‐free survival (RFS), and recurrence pattern were compared between lobectomy and segmentectomy in each group. The selection of the surgical procedure was also examined.

### Statistical analysis

Descriptive statistics were used to assess the patients' demographic characteristics and outcomes. Normally distributed continuous data were expressed as median values. Survival was calculated using the Kaplan–Meier method, and differences in the survival were assessed using a log‐rank analysis. Comparisons among all parameters were analyzed using the Student's *t*‐test. All data were analyzed using the EZR software program (Saitama Medical Center, Jichi Medical University, Saitama, Japan).^9^ This analysis was approved by the Institutional Review Board of Seirei Mikatahara General Hospital (approval no.: 21‐02). Informed consent was obtained from each patient before the examinations and the contents of this study were disclosed at our hospital.

## RESULTS

### Patient characteristics in the ROC analysis

Table 1 shows the patient characteristics of the ROC analysis population. A total of 270 cases were analyzed (stage 0, *n* = 29; stage IA1, *n* = 118; and stage IA2, *n* = 123). Lobectomy and segmentectomy were performed in 174 and 96 cases, respectively. Regarding the pathological stage, most of the cases were stage IA (79%), and 21% were more advanced than stage IB, contrary to preoperative expectations. The histological types were as follows: adenocarcinoma, *n* = 240; squamous cell carcinoma, *n* = 27; and other, *n* = 3. Pathologically invasive carcinoma was found in 108 cases. There were 65 (24.0%) cases with lymphatic invasion, 85 (31.5%) with vascular invasion, 21 (7.7%) with lymph node metastasis, 37 (13.7%) with micropapillary carcinoma, and 23 (8.5%) with STAS. Among the cases with lymph node metastasis, 16 were classified N1 and five were classified as N2.

**TABLE 1:** Patient characteristics in this study

Characteristic	N (%)
Patients	270
Sex	
Male	145 (53.7%)
Female	125 (46.3%)
Median age ‐ years [range]	67 [30–88]
cStage (ver. 8)	
O	29 (10.7%)
IA1	118 (43.7%)
IA2	123 (45.6%)
Surgery	
Lobectomy	174 (64.4%)
Segmentectomy	96 (35.6%)
pStage (ver. 8)	
O	42 (15.5%)
IA1	88 (32.6%)
IA2	70 (26.0%)
IA3	13 (4.8%)
IB	31 (11.5%)
IIB	17 (6.3%)
IIIA	7 (2.6%)
IIIB	1 (0.3%)
IVA	1 (0.3%)
Histology	
Adenocarcinoma	240 (88.9%)
Squamous cell carcinoma	27 (10.0%)
Others	3 (1.1%)
Invasive	108 (40.0%)
Ly (+)	65 (24.0%)
V (+)	85 (31.5%)
LN meta (+)	21 (7.7%)
Micropapillary (+)	37 (13.7%)
STAS (+)	23 (8.5%)

Abbreviations: LN meta, lymph node metastasis; Ly, lymphatic invasion; STAS, spread through air space; V, vascular invasion.

### Best predictors and comparison of the prognosis

**FIGURE 3:**
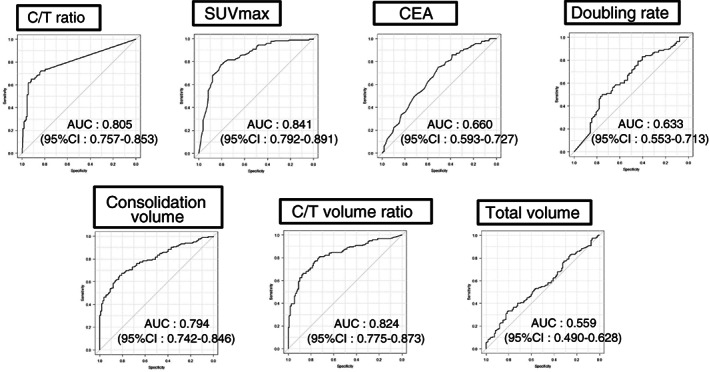
Pathologically noninvasive cancers and the receiver operating curve (ROC) curve for each factor

Figure 3 shows the pathologically noninvasive cancers and the ROC curve for each factor. AUCs were compared. The AUC of the control C/T ratio was 0.805, while the SUVmax was 0.841 and the C/T volume ratio was 0.824; thus showing the C/T ratio showed superior results. Therefore, we set the cutoff values for the three factors and compared their accuracy.

Table 2 shows the cutoff values and sensitivity of these three factors when the maximum cutoff value that showed 0.97 specificity was set with reference to the results of the analysis of JCOG0201. The C/T volume ratio, with a cutoff value of 0.22, was found to be the best factor (sensitivity = 0.395).

**TABLE 2:** Cutoff value and sensitivity to achieve 0.97 specificity

	Cutoff value	Sensitivity
C/T ratio	0.26	0.29
SUVmax	0.5	0.203
C/T volume ratio	0.22	0.395

Abbreviations: C/T ratio, consolidation to tumor ratio; C/T volume ratio, consolidation to tumor volume ratio; SUVmax, maximum standardized uptake value.

### Usefulness of the C/T volume ratio derived from the 3D imaging analysis

**FIGURE 4:**
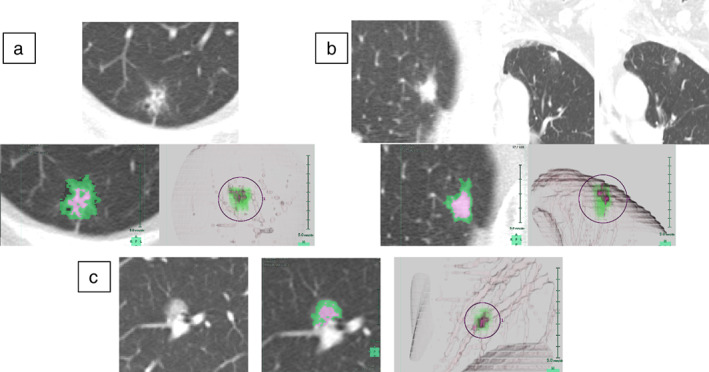
Computed tomography (CT) images and 3D imaging analysis of characteristic cases. A solid component is present in the spots (a). It is difficult to visualize the maximum solid site and ground‐glass opacities (GGO) in a simple single plane image (b). The lesion was a pure GGO on CT but the Synapse Vincent software program recognized a slight difference in the gradation of the CT value and it was judged that there was a solid component (c)

The number of cases in which the C/T volume ratio was superior to the C/T ratio when the cutoff values in Table 2 were used (C/T ratio > 0.26 and C/T volume ratio < 0.22 among patients with pathologically noninvasive cancer) was 26, which was approximately 10% of the total population of the analysis. In CT images in which a solid component was present in spots (Figure 4(a)) and cases in which it was difficult to visualize the site with maximal solid and ground‐glass opacity (GGO) in a simple single plane image (axial, coronal, or sagittal) (Figure 4(b)), the C/T volume ratio was superior to the C/T ratio. It was found that a more accurate ratio could be calculated by evaluating not only the maximum diameter of the GGO region and solid region but also the entire tumor in 3D; this showed a close correlation with the C/T volume ratio in patients with pathologically noninvasive cancer.

Conversely, there were only five cases in which the C/T ratio was superior to the C/T volume ratio (C/T ratio < 0.26 and C/T volume ratio > 0.22 among pathologically noninvasive cancers). In all cases, the lesion was on CT was a pure GGO, and only when Synapse Vincent recognized a slight difference in the gradation of the CT value was it judged that there was a solid component (Figure 4(c)). All of these cases were pathologically diagnosed as adenocarcinoma in situ.

### Prognostic analysis

**FIGURE 5:**
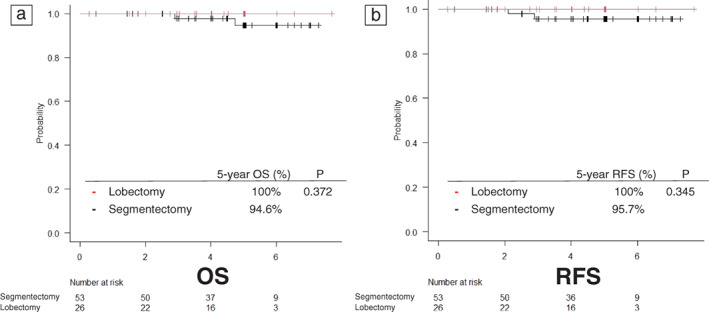
Kaplan–Meier curves for a consolidation to tumor (C/T) volume ratio of <0.22. The 5‐year overall survival (OS) curves (a) and 5‐year recurrence‐free survival (RFS) curves (b) of the two groups

**FIGURE 6:**
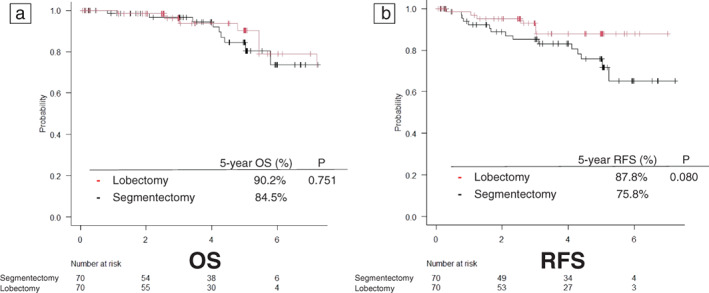
Kaplan–Meier curves in the propensity score‐matched population for a consolidation to tumor (C/T) volume ratio of >0.22. The 5‐year overall survival (OS) curves (a) and 5‐year recurrence‐free survival (RFS) curves (b) of the two groups

Using the C/T volume ratio as an index, we analyzed the OS, RFS, and recurrence pattern in the lobectomy and segmentectomy groups. The background characteristics of patients with a C/T volume ratio of <0.22 was generally consistent between the two groups. Regarding the pattern of recurrence, there was no local recurrence or locoregional recurrence, and no difference was observed between the two groups (Table 3). The analysis showed no significant difference in either OS or RFS (Figure 5). Propensity score matching was performed due to bias in the background factors of patients with a C/T volume ratio of >0.22. In this analysis, regarding the pattern of recurrence, there was no local recurrence in either group, but the recurrence and locoregional recurrence rates tended to be higher in the segmentectomy group (Table 4). Although there was no significant difference in OS, there was a 12% difference in the 5‐year RFS rate, which is a surrogate for OS, with the segmentectomy group showing an inferior result (Figure 6).

**TABLE 3:** Characteristics of patients with a C/T volume ratio of <0.22

	C/T volume ratio < 0.22 (*n* = 79 [29%])		
	Lobectomy (*n* = 26)	Segmentectomy (*n* = 53)	*p*‐value
Sex			
Male	9 (34.6%)	26 (49.1%)	0.241
Female	17 (65.4%)	27 (50.9%)	
Median age ‐ years	63.7	63.4	0.919
cStage (ver. 8)			
O	9 (34.6%)	23 (43.4%)	0.645
IA1	17 (65.4%)	29 (54.7%)	
IA2	0 (0%)	1 (1.9%)	
pStage (ver. 8)			
O–I	26 (100%)	53 (100%)	1
II–IV	0 (0%)	0 (0%)	
Histology			
Adenocarcinoma	26 (100%)	53 (100%)	1
Squamous cell carcinoma	0 (0%)	0 (0%)	
Others	0 (0%)	0 (0%)	
Invasive	3 (11.5%)	1 (1.9%)	0.102
SUVmax	0.95	0.74	0.254
Recurrence	0 (0%)	1 (1.9%)	1
Local recurrence^a^	0 (0%)	0 (0%)	1
Locoregional recurrence^b^	0 (0%)	0 (0%)	1

Abbreviation: C/T volume ratio, consolidation to tumor (C/t) volume ratio; SUVmax, maximum standardized uptake value.

^a^ Surgical margin recurrence.

^b^ Recurrence at the surgical margin, ipsilateral hemithorax, or regional lymph nodes.

**TABLE 4:** Characteristics of patients with a C/T volume ratio of >0.22

	C/T volume ratio >0.22 (*n* = 191 [71%])		
	Lobectomy (*n* = 121)	Sublobar resection (*n* = 70)	*p*‐value
Sex			
Male	76 (62.8%)	37 (52.9%)	0.39
Female	45 (37.2%)	33 (47.1%)	
Median age ‐ years	67.6	69.8	0.07
cStage (ver. 8)			
O	2 (1.6%)	0 (0%)	0.136
IA1	35 (28.9%)	31 (44.3%)	
IA2	84 (69.5%)	39 (55.7%)	
pStage (ver. 8)			
O–I	109 (90.1%)	69 (98.6%)	0.001
II–IV	12 (9.9%)	1 (1.4%)	
Histology			
Adenocarcinoma	111 (91.8%)	56 (80.0%)	0.579
Squamous cell carcinoma	8 (6.6%)	13 (18.6%)	
Others	2 (1.6%)	1 (1.4%)	
Invasive	80 (66.1%)	26 (37.1%)	<0.001
SUVmax	3.99	2.3	0.001

	Propensity score‐matched		
	C/T volume ratio > 0.22 (*n* = 140)		
	Lobectomy (*n* = 70)	Sublobar resection (*n* = 70)	*p*‐value
Sex			
Male	32 (45.7%)	37 (52.9%)	0.499
Female	38 (54.3%)	33 (47.1%)	
Median age ‐ years	68.3	69.8	0.335
cStage (ver. 8)			
O	1 (1.4%)	0 (0%)	0.224
IA1	23 (32.9%)	31 (44.3%)	
IA2	46 (65.7%)	39 (55.7%)	
pStage (ver. 8)			
O–I	69 (98.6%)	69 (98.6%)	1
II–IV	1 (1.4%)	1 (1.4%)	
Histology			
Adenocarcinoma	66 (94.3%)	56 (80.0%)	0.062
Squamous cell carcinoma	3 (4.3%)	13 (18.6%)	
Others	1 (1.4%)	1 (1.4%)	
Invasive	26 (37.1%)	26 (37.1%)	1
SUVmax	2.39	2.3	0.794
Recurrence	3 (4.3%)	10 (14.3%)	0.077
Local recurrence^a^	0 (0%)	0 (0%)	1
Locoregional recurrence^b^	1 (1.4%)	5 (7.1%)	0.209

Abbreviation: C/T volume ratio, consolidation to tumor (C/T) volume ratio; SUVmax, maximum standardized uptake value.

^a^ Surgical margin recurrence.

^b^ Recurrence at the surgical margin, ipsilateral hemithorax, or regional lymph nodes.

## DISCUSSION

To the best of our knowledge, there have been no reports which have demonstrated the relationship between parameters obtained from 3D imaging analyses and pathological malignancies. In this study, the C/T volume ratio was found to be a better predictor of pathologically noninvasive cancer than the C/T ratio. In clinical practice, the CEA and SUVmax are also used as reference findings to select the surgical procedure. A significant correlation between the CEA level and both nodal involvement and survival in stage I NSCLCs has been reported,^10^ but judging from the ROC curve in this study, it was difficult to say that CEA has a good correlation with pathological factors associated with a poor prognosis. This may be due to the breakdown of the C/T ratio within the subjects who were analyzed, and lobectomy should still be considered for patients with high CEA values. However, it should be noted that there was no correlation between low CEA pathological factors associated with a good prognosis. Solid tumors with an SUVmax of ≥2.5 have been reported be associated with a high risk of recurrence, and the indications for reduction surgery must be carefully considered in patients with this factor.^11^ Furthermore, Tsutani et al. defined SUVmax <1.5 as an indication for segmentectomy.^12^ In this study, the SUVmax had very high specificity for pathologically noninvasive carcinoma, but did not have very high sensitivity. This is in line with previous reports.^13^ Hattori et al. compared 3‐year locoregional recurrence‐free survival in stage IA adenocarcinoma between sublobar resection and lobectomy and reported that sublobar resection was associated with significantly worse outcomes when the SUVmax was high.^14^ Therefore, it should only be used as a reference when the SUVmax is high. In other words, a low SUVmax does not necessarily indicate a pathologically noninvasive cancer.

When the C/T volume ratio was <0.22, which almost guarantees pathologically noninvasive cancer (specificity >0.97), there was no difference in OS, RFS, or the recurrence pattern between the lobectomy and segmentectomy groups. When the C/T volume ratio was >0.22, the sample size was reduced by propensity score matching, and there was no significant difference, but segmentectomy showed a clear tendency toward an association with inferior RFS. This tendency became more pronounced as the cutoff value of the C/T volume ratio was increased; thus sublobar resection with curative intent should not be indicated.

There were many cases with a large difference in values between the C/T ratio and the C/T volume ratio, and although similar, they are considered be completely different indexes. In JCOG0802, when the C/T ratio was used, there was no difference in RFS between the lobectomy and segmentectomy groups. In this study, the use of the C/T volume ratio helped to better discriminate between pathologically invasive and pathologically noninvasive carcinoma, which may have caused the difference in RFS. Regarding OS, it is difficult to evaluate OS in general because it can change greatly depending on the contents of treatment after recurrence; however, at least in this study, there was no result suggesting that segmentectomy was associated with superior OS in any group.

Although this study was started from the viewpoint of local control, there was no case of local recurrence in the analysis group of our department, and it was impossible to compare local recurrence between the surgical techniques. Our department has been using Synapse Vincent since 2013, and local control may be possible by selecting a surgical procedure that can secure a firm margin, even if there are many solid components. It has been reported that the rate of STAS was high in micropapillary type and solid type tumors, and the farthest lesion was 1.7 cm from the tumor.^15^ Therefore, as indicated in the National Comprehensive Cancer Network guidelines and previous reports, we believe that a margin of 2 cm or more is desirable.^16^ In addition, the smaller the lesion size, the greater the error between the measurement in the index, as represented by the ratio. We think that the conventional C/T ratio is measured manually, which tends to result in large errors between operators. On the other hand, semi‐automatic measurement using a 3D imaging analysis software program can reduce any artificial measurement error and reflect more accurate values.

The present study was associated with some limitations, including its single‐center, retrospective design. Second, in this analysis, lymph node metastasis was used as an evaluation factor for invasive cancer. In our department, when segmentectomy is performed, an accurate evaluation is not possible because it is limited to group 1 dissection. Third, the possibility of measurement error by Synapse Vincent cannot be excluded. This software also recognizes the blood vessels running inside the tumor as solid components. However, it is possible to unify the differences in the measured values ​​between individuals, even in the same case when the 3D imaging analysis software program was used for the calculation. Also, the compatibility with 3D imaging analysis software programs other than the Synapse Vincent is unknown.

In conclusion, in comparison to the conventional C/T ratio, the C/T volume ratio may be a better preoperative predictor of pathologically noninvasive cancers that can be used to determine whether or not to perform sublobar resection. As for the selection of surgical procedure, we should be cautious about indications for sublobar resection, because the risk of recurrence increases with sublobar resection in patients with many solid components on 3D images, even if they have peripheral lung cancer that is small in size.

## AUTHOR CONTRIBUTIONS

Hiroyuki Tsuchida: Conceptualization, writing – original draft, formal analysis, investigation, methodology, supervision. Masayuki Tanahashi: Methodology and supervision. Eriko Suzuki: Investigation. Naoko Yoshii: Investigation. Takuya Watanabe: Investigation. Shogo Yobita: Investigation. Suiha Uchiyama: Investigation. Kensuke Iguchi: Investigation. Minori Nakamura: Investigation. Takumi Endo: Investigation. All authors discussed the results and approved the final version of the manuscript.

## CONFLICT OF INTEREST

The authors declare that they have no competing interests.
